# Three years follow-up of screen-detected diabetic and non-diabetic subjects: who is better off? The ADDITION Netherlands study

**DOI:** 10.1186/1471-2296-9-67

**Published:** 2008-12-16

**Authors:** Paul GH Janssen, Kees J Gorter, Ronald P Stolk, Mehmet Akarsubasi, Guy EHM Rutten

**Affiliations:** 1 Julius Center for Health Sciences and Primary Care, University Medical Center, Utrecht, the Netherlands; 2 Department of Epidemiology, University Medical Center, Groningen, the Netherlands

## Abstract

**Background:**

People with non-diabetic hyperglycaemia might be at risk of lacking adequate control for cardiovascular risk factors. Our aim was to determine the extent of health care utilization and provision in primary care and to evaluate the risk of cardiovascular disease in persons with an elevated risk score in a stepwise diabetes screening programme.

**Methods:**

A total of 56,978 non-diabetic patients, aged 50–70 years, from 79 practices in the Netherlands were invited to participate in a screening programme starting with a questionnaire. Those with an elevated score, underwent further glucose testing. Screened participants with type 2 diabetes (*n *= 64), impaired glucose tolerance (IGT) (*n *= 62), impaired fasting glucose (IFG) (*n *= 86), and normal glucose tolerance (NGT) (*n *= 142) were compared after three years regarding use of medication, care provider encounters and occurrence of CVD.

**Results:**

In all glucose regulation categories cardiovascular medication was prescribed more frequently during follow-up with the strongest increase in diabetic patients. Number of practice visits was higher in diabetic patients compared to those in the other categories. Glucose, lipids, and blood pressure were measured most frequently in diabetic patients. Numbers of cardiovascular events in participants with NGT, IFG, IGT and diabetes were 16.7, 32.6, 17.3 and 15.7 per 1,000 person-years (non significant), respectively.

**Conclusion:**

After three years of follow-up, screened non-diabetic participants with an elevated risk score had cardiovascular event rates comparable with diabetic patients. Screened non-diabetic persons are at risk of lacking optimal control for cardiovascular risk factors while screen-detected diabetic patients were controlled adequately.

## Background

Type 2 diabetes is an important health problem and the costs associated with its treatment are increasing [1]. Diabetes is a significant cause of cardiovascular morbidity and mortality. In addition, patients with 'pre-diabetes' (impaired fasting glucose (IFG) and impaired glucose tolerance (IGT)) are at an increased risk for cardiovascular diseases (CVD) and higher medical care costs [2,3]. Awareness of the cardiovascular risk of impaired glucose regulation among general practitioners is relatively low [4]. As a result people with non-diabetic hyperglycaemia might be at risk of lacking adequate control for their cardiovascular risk factors. Participants in a diabetes screening programme who had an elevated score on a risk questionnaire already had a high mortality risk whether or not subsequent diagnostic testing classified them to have diabetes [5,6]. This suggests that it may be of greater benefit to intervene in the larger population with an increased cardiovascular risk rather than only in the smaller group of screen-detected people with diabetes.

Screening for diabetes is recommended by the American Diabetes Association, although definitive evidence of its effectiveness is lacking [7,8]. Diabetic patients identified in a screening programme typically receive treatment to control cardiovascular risk factors while people in whom the diagnosis of diabetes could not be established are usually reassured. However, little is known about the natural history of screened people with an elevated risk score in a diabetes screening programme. Therefore, it is disputable whether reassurance is justified.

The aim of the present study was to determine the use of medication and the frequency of care provider encounters after three years of follow-up of diabetic and non-diabetic persons with an elevated risk score in a population-based screening programme. In addition, we evaluated their risk of CVD during three years of follow-up.

## Methods

This study is part of the ADDITION study (Anglo-Danish-Dutch Study of Intensive Treatment in People with Screen-Detected Diabetes in Primary Care), a randomised controlled trial consisting of a screening and an intervention study [9]. Screen-detected diabetic patients in practices in the intervention arm of the ADDITION study undergo a multifactorial treatment according to strict targets to reduce their cardiovascular risk. Those in the control practices receive routine care according to the guidelines from the Dutch College of General Practitioners [10]. The study was approved by the medical-ethical committee of the University Medical Center Utrecht. Participants gave written informed consent.

In the Netherlands, we performed a population-based screening for type 2 diabetes from May 2002 to April 2004 [11]. The screening algorithm in the three countries was comparable [12]. In the Netherlands, the screening started with a self-completed questionnaire, mailed to 56,978 non-diabetic, predominantly Caucasian, registered patients, aged 50–70 years, from 79 general practices in the southwestern region of the Netherlands. This validated questionnaire contained questions about age, gender, body mass index, family history of diabetes, frequent thirst, use of antihypertensive medication, shortness of breath, claudication, and cycling [13].

Participants were invited for further diagnostic testing including the OGTT if they scored above threshold on the questionnaire [11]. All subjects were classified as having either type 2 diabetes, IGT, IFG or normal glucose tolerance (NGT) according to the 1999 WHO criteria [14]. Diagnosis of diabetes was based on two diabetic glucose values [11]. Eventually, we detected 586 new diabetic patients. A letter with the test results was handed over to all participants and the implications were discussed. In addition, participants' general practitioners were informed.

### Study population

For the present study, all participants with a risk score above threshold from 24 practices, screened from May to October 2002, were eligible. Screen-detected diabetic patients from practices in the intervention arm of the ADDITION study were excluded. Finally, 392 participants were included: all 70 diabetic patients in the control arm of the study, all 66 persons with IGT, all 96 persons with IFG and 160 people with normal glucose tolerance (NGT), randomly selected out of all 3,258 participants with NGT.

Of these people, 38 were not found in the patient files in the practices, partially because of changing of practice or address since screening, partially for reasons unknown. Thus, we collected data at baseline and after three years from 354 participants in four categories of glucose regulation: participants with type 2 diabetes receiving usual care (*n *= 64), with IGT (*n *= 62), with IFG (*n *= 86), and with NGT (*n *= 142).

### Measurements

Baseline and follow-up data were collected by one of the authors (MA) from the medical records of the patients in the practices. All practices gave approval of investigation of their patients' medical records. Incidence of diabetes, all cause mortality and cardiovascular morbidity (non-fatal myocardial infarction, non-fatal stroke, amputation, hospitalisation for angina pectoris, hospitalisation for congestive heart failure, coronary revascularisation, peripheral vascular events) were recorded with date of diagnoses. Numbers of care provider encounters, referrals to a medical specialist (cardiologist, internist, neurologist), and glucose, lipids and blood pressure measurements during follow-up, were derived from patients' medical records. Use of medication (lipid lowering drugs, antihypertensive agents, acetylsalicylic acid) was assessed at baseline and after three years (issued data). Values of anthropometric (BMI and blood pressure) and biochemical (glucose, cholesterol, HDL-cholesterol, LDL-cholesterol, and triglycerides) characteristics, derived from the patient records, were determined at baseline and after three years. All practices collaborate with the same regional laboratory (SHL Center for Diagnostic Support in Primary Care, Etten-Leur, The Netherlands) for all biochemical tests.

### Statistical analyses

Data were analyzed applying the SPSS statistical package (version 11.0). Gender and age at baseline were compared between the different diagnostic categories using the χ^2 ^test for dichotomous variables and ANOVA for continue variables, respectively. Cumulative occurrence of diabetes in the non-diabetic categories during follow-up was compared using the χ^2 ^test. Health care encounters were analyzed using the Mann-Whitney test. Differences in use of medication were examined with the χ^2 ^test. Paired-samples *t*-tests were used to compare baseline with follow-up antropometric and biochemical measurements within each diagnostic category. Cardiovascular events and death were calculated per diagnostic category per 1,000 person-years of follow-up. Cox regression analysis with age and gender entered as covariates in the model was used to examine whether the diagnostic categories have different risks regarding mortality and cardiovascular morbidity. A *P *value < 0.05 was considered significant.

## Results

Characteristics of participants in the different diagnostic categories are presented in Table 1. A total of 24 subjects (8.2%) in the non-diabetic categories developed diabetes. Development of diabetes was highest among participants with IGT (16.1%).

**Table 1:** Characteristics of participants in different categories of glucose regulation

	**NGT** **(*n *= 142)**	**IFG** **(*n *= 86)**	**IGT** **(*n *= 62)**	**type 2 diabetes** **(*n *= 64)**
Age (years) (at baseline)	61.5 ± 5.9	61.0± 5.4	61.7 ± 5.8	61.0 ± 5.4
Gender (male %)	39.4*	53.5	48.4	60.9
Incidence of diabetes *n *(%)	6 (4.2)†	8 (9.3)	10 (16.1)	-

Data are presented as mean ± SD except where otherwise specified.

* NGT significantly different from IFG and type 2 diabetes; † NGT significantly different from IFG and IGT.

Use of medication at baseline and after three years is shown in Table 2. At baseline, use of cardiovascular medication in all diagnostic categories was comparable. Overall, in all diagnostic categories an increase in use of medication was found. Both the increase in use of lipid lowering drugs and antihypertensive agents were strongest in participants with diabetes (35.9% and 42.2%, respectively). In addition, Table 2 shows a significant decrease in blood pressure in the follow-up period in all categories except in persons with NGT. Furthermore, we found in type 2 diabetic patients a significant improvement in levels of total cholesterol and LDL-cholesterol (total cholesterol (mean ± SD) 5.6 ± 1.2 mmol/l (at baseline) and 4.7 ± 1.0 mmol/l (after one year); LDL-cholesterol 3.6 ± 1.0 mmol/l and 2.9 ± 0.8 mmol/l, respectively). HDL-cholesterol and triglycerides also improved but not significantly: HDL-cholesterol changed from 1.0 ± 0.3 mmol/l at baseline to 1.1 ± 0.3 mmol/l and triglycerides from 2.4 ± 2.5 mmol/l to 1.6 ± 0.8 mmol/l. BMI in diabetic patients decreased from 31.0 ± 5.2 kg/m^2 ^to 29.1 ± 5.2 kg/m^2^. In the non-diabetic categories, we did not find enough measurements of lipids and weight in the patient records, which made a comparison with the type 2 diabetes category impossible.

**Table 2:** Characteristics in different categories of glucose regulation at baseline and after three years

	**NGT (*n *= 142)**		**IFG (*n *= 86)**		**IGT (*n *= 62)**		**type 2 diabetes (*n *= 64)**	
	**T0**	**T3**	**T0**	**T3**	**T0**	**T3**	**T0**	**T3**

*Use of medication*								
Antihypertensive drugs (%)	21.1	42.3	31.4	48.8	41.9	66.1	31.3	67.2*
Lipid lowering drugs (%)	14.8	32.4	17.4	27.9	14.5	32.3	14.1	56.3†
Acetylsalicylic acid (%)	9.2	19.7	16.3	22.1	11.3	22.6	9.4	15.6
								
*Anthropometric characteristics*								
SBP (mm Hg)	147 ± 33	146 ± 20	151 ± 24	145 ± 23‡	160 ± 24	143 ± 21‡	156 ± 21	140 ± 13‡
DBP (mm Hg)	83 ± 20	84 ± 10	86 ± 11	82 ± 10‡	89 ± 12	80 ± 11‡	89 ± 9	80 ± 7‡

Data are presented as mean (± SD) except where otherwise specified.

T0, at baseline; T3, after three years; SBP, systolic blood pressure; DBP, diastolic blood pressure.

* Difference in use of medication at T3 and T0 in type 2 diabetes category significantly different from differences in NGT and IFG categories; † difference in use of medication at T3 and T0 in type 2 diabetes category significantly different from differences in all other categories; ‡ difference in mean in diagnostic category at T0 and T3 significant.

During follow-up diabetic patients contacted significantly more often the practice (median 33.5, interquartile range 22.3–46.0) (Table 3). Frequencies of blood pressure, glucose and lipids measurements were highest in type 2 diabetic patients. Persons with IGT scored higher in this respect than people with IFG and NGT. The numbers of referrals to a the cardiologist, internist and neurologist did not differ between the different diagnostic categories.

**Table 3:** Health care encounters in different categories of glucose regulation over three years

	**NGT** **(*n *= 142)**	**IFG** **(*n *= 86)**	**IGT** **(*n *= 62)**	**type 2 diabetes** **(*n *= 64)**
Care provider encounters	20.0 (11.0–34.0)	23.0 (9.0–34.0)	26.0 (15.0–43.5)*	33.5 (22.3–46.0)†
Blood pressure measurements	2.0 (0–5.0)	2.0 (0–5.0)	4.0 (1.0–6.0)*	5.0 (3.0–7.0)†
Glucose measurements	1.0 (0–2.0)	1.0 (0–3.0)	2.0 (1.0–4.0)*	13.0 (7.3–14.8)†
Lipid measurements	0.5 (0–2.0)	1.0 (0–2.0)	1.0 (0–2.0)‡	3.0 (2.0–4.0)†
Referrals to specialist	2.0 (1.0–4.0)	2.0 (1.0–4.0)	2.0 (1.0–4.0)	2.0 (0–3.0)

Data are presented as median (interquartile range).

* IGT significantly different from all other categories; † type 2 diabetes significantly different from all other categories; ‡ IGT significantly different from NGT and type 2 diabetes.

Numbers of cardiovascular events and death in participants with NGT, IFG, IGT and diabetes were 16.7, 32.6, 17.3 and 15.7 per 1,000 person-years, respectively. Age and gender adjusted differences between the diagnostic categories were not statistically significant (Table 4).

**Table 4:** Cardiovascular events and death from all causes in different categories of glucose regulation

	**NGT** **(*n *= 142)**		**IFG** **(*n *= 86)**		**IGT** **(*n *= 62)**		**type 2 diabetes** **(*n *= 64)**	
Person-years of follow-up	420.4		245.4		173.5		191.5	
Event	**n**	**per 1000 person-years**	**n**	**per 1000 person-years**	**n**	**per 1000 person-years**	**n**	**per 1000 person-years**
Death (all causes)	1	2.4	2	8.1	0	0	1	5.2
Non-fatal MI	0	0	1	4.1	0	0	0	0
Non-fatal stroke	1	2.4	0	0	1	5.8	1	5.2
Amputation	0	0	0	0	0	0	0	0
Hospitalisation AP	3	7.1	0	0	0	0	0	0
Hospitalisation CHF	0	0	0	0	0	0	1	5.2
Coronary revascularisation	0	0	2	8.1	1	5.8	0	0
Peripheral vascular events	2	4.8	3	12.2	1	5.8	0	0

Total	7	16.7	8	32.6	3	17.3	3	15.7*

* Differences between categories not significant.

## Discussion

In this study we followed people in different glucose regulation categories during three years, who all had an elevated risk score in a diabetes screening programme. In the follow-up period almost one in six participants with IGT developed diabetes, whereas almost one in ten people with IFG did. In all glucose regulation categories cardiovascular medication was prescribed more frequently at end of follow-up compared to baseline with the strongest increase in diabetic patients. Numbers of practice visits and cardiovascular risk factors measurements were highest in diabetic participants followed by those with IGT. Apparently, people with diabetes were treated more intensively and down to target, compared to high risk non-diabetic individuals, who are eligible for preventive measures as well. Our findings indicate that there is a missed opportunity for effective preventive care for non-diabetic high risk individuals in general practice. Screened non-diabetic persons with an elevated risk score appeared to have subsequent cardiovascular events at a level comparable with diabetic patients.

Most other studies also showed the progression rate to type 2 diabetes to be higher in people with IGT than in those with IFG with the highest rate in people with both IGT and IFG (combined-IGT) [15-18]. The incidence rate of diabetes in persons with IGT and IFG in our study is rather low compared with findings in other studies. The progression rate in the ADDITION Denmark study was higher. Participants with IGT and IFG in this study were invited for re-examination after one year and diagnosis of diabetes was based on one diabetic glucose value [12]. In our study diabetes was diagnosed during regular practice visits which may be less sensitive. In the Dutch Hoorn study, the cumulative incidence of diabetes was 64.5% [16]. However, these were participants with combined-IGT, the mean follow-up was 5.8–6.5 years and participants were aged 50 to 75 years at baseline.

Although IGT is more strongly associated with cardiovascular disease than IFG [19-22], in this study most cardiovascular events occurred in participants with IFG and least in diabetic patients. However, differences between the four diagnostic categories were not statistically significant. It may be assumed that the relatively low event rate in diabetic patients is associated with the provision of structured diabetes care following the diagnosis of diabetes, while participants in the other diagnostic categories were lacking such care. That said, non-diabetic persons are being controlled for hypertension and dyslipidaemia according to Dutch national guidelines, but this is done less systemically than in those with diabetes. Prevention or delay of onset of type 2 diabetes is possible in individuals with IGT, either by lifestyle changes or by pharmacological interventions [23,24]. However, awareness of the clinical significance of IGT among general practitioners is relatively low [4]. General practitioners need to be motivated and resourced to perform preventive strategies [25]. These findings seem consistent with our observation that diabetic patients contacted the practice more frequently compared with non-diabetic participants. In addition, it is understandable that the increase in use of cardiovascular medication after three years was highest in diabetic patients. In the non-diabetic categories the use of cardiovascular medication also increased, although this increase was considerably less. We should realise that all participants had an elevated risk score indicating an unfavourable cardiovascular risk profile. The 1999 guidelines from the Dutch College of General Practitioners recommended yearly glucose testing in people with increased (but non-diabetic) fasting glucose levels. However, it is not solely an issue to test people with impaired glucose regulation for diabetes. It seems more appropriate to control them for cardiovascular risk factors.

Some limitations of this study need to be addressed. Firstly, screen-detected diabetic patients who were treated intensively in the intervention arm of the ADDITION study were excluded. Care providers in these practices might have developed during the study an increased 'diabetes awareness' possibly resulting in a changed attitude towards people with IFG or IGT. However, the number of health care encounters of non-diabetic participants in intervention and control practices did not differ significantly (data not shown). Secondly, we could not compare plasma lipids between the different diagnostic categories simply because of not finding enough data of the non-diabetic participants in their records. Nevertheless, it is unlikely that the plasma lipid levels in the non-diabetic participants have improved to the same extent as in the diabetic patients, since the increase in use of lipid lowering drugs during follow-up was substantially lower in participants without diabetes. Thirdly, this sub-study of the ADDITION study was not designed prospectively to investigate differences in the occurrence of cardiovascular events between the glucose regulation categories. No power analysis was performed. The relatively small size of the diagnostic categories may account for the non-significant differences between the groups. The small sample size combined with low event rate might have lead to type 2 errors. Therefore, it is not allowed to draw firm conclusions regarding the non-significance of the CVD risk between the groups. Despite this, we feel that the occurrence of CVD is consistent with the trend in our other observations. Still, our purpose primarily was to examine whether high risk non-diabetic participants receive adequate control for CVD risk factors after the screening. Fourthly, data collection was not done blinded. However, it may be assumed that this did not introduce bias because data were derived retrospectively from patients' files at the practices and in all practices record-keeping of the patients was computerized.

Our data indicate that screened non-diabetic people with an elevated score on a questionnaire, mainly consisting of cardiovascular items, who do not receive structured care following the screening should not be reassured only because of not having diabetes. People diagnosed with diabetes in a screening programme seem better off regarding their CVD risk than those in whom diagnosis of diabetes could not be established.

## Conclusion

The diabetes risk questionnaire identified people with increased cardiovascular risk. Screened participants with an elevated risk score but without diabetes are at risk of lacking optimal medical care in order to control for cardiovascular risk factors. Moreover, our data demonstrated that adequate control for cardiovascular risk factors in asymptomatic diabetic patients identified by screening is achievable in general practice. GPs might be relatively unaware of the cardiovascular risk of screened persons with impaired glucose regulation, they are not neglecting the importance of treating screen-detected diabetic patients. Our findings indicate that screening for an unfavourable cardiovascular risk profile is preferable to screening for diabetes only.

## Abbreviations

CVD: cardiovascular disease; IFG: impaired fasting glucose; IGT: impaired glucose tolerance; NGT: normal glucose tolerance; OGTT: oral glucose tolerance test; RBG: random blood glucose; WHO: World Health Organization.

## Competing interests

The authors declare that they have no competing interests.

## Authors' contributions

PJ drafted the manuscript. MA collected data were from the medical records of the patients in the practices. All authors helped to refine the analysis, contributed to the final version of the paper and approved the final manuscript.

## Pre-publication history

The pre-publication history for this paper can be accessed here:


